# Associations between early life adversity and the development of gray matter macrostructure and microstructure

**DOI:** 10.1017/S0033291725102651

**Published:** 2025-12-18

**Authors:** Anders Lillevik Thorsen, Florence Friederike Boehmisch, Dag Alnæs, Andreas Dahl, Lars T. Westlye, Olga Therese Ousdal

**Affiliations:** 1 Bergen Center for Brain Plasticity, Haukeland University Hospital, Bergen, Norway; 2 Centre for Crisis Psychology, University of Bergen, Bergen, Norway; 3 Department of Anatomy and Neurosciences, Amsterdam UMC, Amsterdam, Netherlands; 4 Department of Biomedicine, University of Bergen, Bergen, Norway; 5 Centre for Precision Psychiatry, Oslo University Hospital & Institute of Clinical Medicine, University of Oslo, Oslo, Norway; 6 Department of Psychology, University of Oslo, Oslo, Norway; 7 KG Jebsen Centre for Neurodevelopmental Disorders, University of Oslo, Oslo, Norway; 8 Department of Radiology, Haukeland University Hospital, Bergen, Norway

**Keywords:** brain development, early life adversity, gray matter, macrostructure, microstructure, trauma

## Abstract

**Background:**

Early life adversity (ELA) is common and cross-sectionally associated with brain gray matter structure, including cortical thickness, cortical surface area, and subcortical volumes in childhood. However, to which degree ELA influences the trajectory of gray matter macrostructural and microstructural development during childhood and adolescence remains largely unexplored.

**Methods:**

We included 6414 participants from the Adolescent Brain Cognitive Development study at ages 9–11, where 1923 were followed to ages 11–13. We used linear mixed-effects models to test for associations between MRI-derived longitudinal measures of gray matter macro- (cortical thickness, surface area, subcortical volume) or microstructure (T1w/T2w ratio) and trauma exposure, parental acceptance, household abuse, and being resilient or susceptible to trauma in terms of developing an internalizing disorder.

**Results:**

At ages 9–11, higher levels of parental acceptance, trauma exposure, and being trauma resilient were associated with lower levels of cortical thickness. In contrast, being trauma susceptible was negatively related to hippocampal volume and cortical surface area. Longitudinally, more parental acceptance at baseline was associated with more cortical thinning between ages 9–11 and 11–13, while more household abuse was associated with less change in T1w/T2w ratio over time.

**Conclusions:**

Parental acceptance and trauma resilience are linked to accelerated pace of apparent cortical thinning in youth aged 9–13 years, while household abuse is associated with slower microstructural development, as reflected by smaller longitudinal changes in the T1w/T2w ratio. Threat and deprivation may be distinctly associated with gray matter developmental trajectories in late childhood.

## Highlights

- Higher levels of parental acceptance and being trauma resilient are associated with a thinner cortex at ages 9–11.
- Experiencing both trauma and an internalizing disorder is linked to smaller hippocampus volume and cortical surface area.
- More parental acceptance predicts faster cortical thinning, while more household abuse predicts slower gray matter microstructural development from ages 9–11 to 11–13.
- Specific indices of early life adversity may be distinctly associated with gray matter development in late childhood.

## Introduction

Experiencing early life adversity (ELA), including trauma, deprivation, and threats, during the formative years of childhood and adolescence, is pervasive and often detrimental for a range of life outcomes (McLaughlin, Weissman, & Bitrán, 2019). More than half of the children in the United States experience a traumatic event by the time they reach adulthood. ELA is associated with poor educational attainment and physical health as well as an increased risk for internalizing mental disorders (McLaughlin, Colich, Rodman, & Weissman, 2020). Understanding how ELA affects brain development during early adolescence may unravel the biological mechanisms linking ELA to the development of psychopathology later in life, as well as differentiate the neural circuitries of resilience to psychopathology following ELA (Masten, Lucke, Nelson, & Stallworthy, 2021).

Childhood and adolescence are periods of considerable brain maturation (Foulkes & Blakemore, 2018), which include overproduction and subsequent pruning of synapses, as well as a continuous increase in axonal myelination (Norbom et al., 2021). These cellular changes may translate into MRI-observable, age-specific changes in gray matter structure. Indeed, normative developmental models suggest a rapid increase in cortical thickness until the age of two, which is then followed by a nonlinear decrease in later childhood and adolescence (Rutherford et al., 2022; Tamnes et al., 2017). Similar patterns have also been observed in several subcortical gray matter regions, where an initial volumetric increase in early childhood is followed by a later flattening or even volumetric decrease until adulthood (Herting et al., 2018; Rutherford et al., 2022). In contrast, cortical surface area continues to expand throughout childhood and early adolescence, and then undergoes subtle decreases thereafter (Tamnes et al., 2017). Notably, ELA may impact both the pace and the trajectory of these brain maturational processes (Foulkes & Blakemore, 2018; McLaughlin et al., 2019).

There are multiple models for understanding the association between ELA and brain development. The cumulative-risk model assumes that all adversities are biologically embedded through similar mechanisms and that there is a dose–response association between ELA and altered brain development (Evans, Li, & Whipple, 2013). The dimensional model differentiates between experiences related to threat, defined as harm or threat of harm to the child, versus deprivation, defined as the lack of age-expected cognitive or emotional stimulation (McLaughlin & Sheridan, 2016). In support of the cumulative model, the number of ELAs has been linked to smaller amygdala, hippocampus, medial, and inferior prefrontal cortex volumes (Pollok et al., 2022) and reduced cortical thickness in the cingulate and middle frontal gyrus (Yang et al., 2023). A study using baseline data from 9720 youths aged 9–11 from the Adolescent Brain Cognitive Development (ABCD) study found that a latent factor for trauma exposure was associated with thinner superior frontal, caudal middle frontal cortices, smaller putamen and amygdala volumes, and thicker isthmus cingulate and posterior cingulate cortices (Jeong et al., 2021).

In support of the dimensional model, a recent study of children aged 5–10 years found threat to be specifically linked to widespread smaller cortical surface area (Machlin et al., 2023). Deprivation was linked to larger cortical thickness in the insula, occipital, parietal, and temporal lobe (Machlin et al., 2023). Threat may also be negatively associated with cortical thickness in the ventromedial PFC, insula, and other parietal, temporal and occipital regions in participants aged 8–17 years (Peverill et al., 2023). However, an important limitation of most existing studies is the lack of longitudinal data, which could illuminate if ELA is linked to the pace of brain development.

In contrast to the numerous studies of ELA and gray matter macrostructure, how ELA relates to the development of gray matter microstructure remains largely unexplored. Indeed, changes in gray matter thickness, surface area, and volume during adolescence may reflect a mixture of synaptic pruning and myelination effects (Grydeland et al., 2019; Norbom et al., 2021). An increasing number of preclinical and human postmortem studies suggest that ELA impairs oligodendrogenesis and myelination (Warhaftig, Almeida, & Turecki, 2023). Indirect and in vivo measures of gray matter myelination can be derived from the ratio between T1w and T2w MRI (T1w/T2w) (Norbom et al., 2021). This is based on the associations between fat, T1w, and T2w signal intensity, the high cholesterol levels of myelin; and the correspondence between the T1w gray matter signal and histologically obtained myelin profiles (Eickhoff et al., 2005; Stüber et al., 2014). While no studies have investigated the T1w/T2w ratio in relation to trauma, threat, or deprivation, T1w/T2w ratio has been associated with socioeconomic status (SES), which is itself associated with ELA (Tooley, Bassett, & Mackey, 2021). For example, higher T1w/T2w ratios in youths from lower SES backgrounds have been reported, suggesting that they show a more myelinated and mature cortex (Norbom et al., 2022). However, other studies have reported mixed results (Boroshok et al., 2023; Weissman et al., 2023). Finally, a study using magnetization transfer found that neighborhood poverty was related to slower myelin growth, suggesting an effect of SES on the pace of gray matter myelination (Ziegler et al., 2020).

ELA is a risk factor for the later development of internalizing disorders (McLaughlin et al., 2020). Internalizing disorders in childhood or adolescence have been associated with both less surface area of frontal, visual, premotor, and parietal cortices (Schmaal et al., 2017), thicker frontal, parietal, temporal, and pre-/postmotor cortices (Yu et al., 2023), and greater prefrontal gray matter volume (Smolker, Snyder, Hankin, & Banich, 2022). However, some individuals appear to be resilient to ELA and do not develop adverse outcomes, including internalizing disorders, despite the exposure to one or several ELAs (Masten et al., 2021; McLaughlin et al., 2020). Notably, trauma-exposed girls who later develop an internalizing disorder may have slower hippocampal volumetric enlargements compared to age-matched resilient girls (Keding et al., 2021), and adolescents with increasingly resilient responses to psychosocial stress may experience increased prefrontal gray matter myelination as measured by magnetization transfer imaging (Hettwer et al., 2024). These findings suggest that being susceptible or resilient to ELA is related to the development of gray matter microstructure and macrostructure in childhood and adolescence. However, this hypothesis requires further investigation in larger samples with longitudinal data.

We investigated whether experiencing ELA influences the trajectory of gray matter macrostructural and microstructural development during the formative years of adolescence. We first tested for associations between baseline regional cortical thickness, subcortical volumes, cortical surface area, and T1w/T2w ratio and trauma exposure, deprivation (operationalized as less parental acceptance and support), and threat (operationalized as household abuse) at ages 9–11. Next, using longitudinal data, we examined the interaction between trauma exposure, parental acceptance, or household abuse at ages 9–11 and subsequent changes in regional cortical thickness, subcortical volumes, cortical surface area, and T1w/T2w ratio between ages 9–11 and 11–13. Based on existing studies, we hypothesized that adolescents experiencing more household abuse would show faster thinning of prefrontal cortical regions, as well as a steeper volumetric increase in the hippocampus and the amygdala, reflecting faster maturation in line with normative models (Colich, Rosen, Williams, & McLaughlin, 2020; Herting et al., 2018; McLaughlin et al., 2019; Tamnes et al., 2017). Finally, we tested whether the trajectory of gray matter macrostructural or microstructural development differed between trauma-resilient and trauma-susceptible individuals, as well as those who neither experienced trauma nor an internalizing disorder.

## Methods

### Participants

The ABCD study is a longitudinal cohort study that follows participants from ages 9–11 to 19–21, investigating how the brain develops throughout this formative period and how biological and environmental factors may impact on the gray and white matter developmental trajectories (Casey et al., 2018). The study recruited participants from 21 sites across the United States, largely through schools, intending to recruit a relatively representative sample of the United States’ population (Garavan et al., 2018). We used tabulated data from Annual Release 4.0 of the ABCD study, which includes demographic, clinical, and MRI data from 11,876 participants at ages 9–11 years and a second MRI at ages 11–13 for 7859 of the participants in the present data release.

### Measures

The ABCD study includes established measures on several forms of ELA meant to capture relevant stressors across multiple domains, using a mix of parent- and child-reported data (Hoffman et al., 2019). For parent-rated measures, we only used information from the primary caregiver due to large amounts of missing data from the secondary caregiver. We used the baseline measures of ELA, which covers ELA exposures up until the age of 9–11 years (the first MRI). Diagnostic criteria for internalizing disorder were assessed using the computerized parental version of the schedule for Affective Disorders and Schizophrenia for School-Age Children-Present and Lifetime version (Kaufman et al., 1997; Kiddie-SADS-PL). This computerized version has been found to have adequate psychometric properties, including good to excellent concordance with clinician-administered versions (Townsend et al., 2020). We also used the post-traumatic stress module of the Kiddie-SADS-PL to estimate the number of experienced traumatic situations. Following the procedures in Keding et al. (2021), we used the Kiddie-SADS-PL to define resiliency or susceptibility to trauma versus normal development. Individuals with an internalizing disorder (i.e. past or present persistent depressive disorder, major depressive disorder, panic disorder, agoraphobia, separation anxiety disorder, social anxiety disorder, generalized anxiety disorder, or post-traumatic stress disorder) who had experienced ≥1 traumatic event were defined as susceptible, while individuals who had experienced ≥1 traumatic event but did not have an internalizing disorder diagnosis were defined as resilient. These groups were compared to participants who neither experienced a traumatic event nor were having an internalizing disorder.

Based on the available measures in the ABCD study, we operationalized threat as reports of household abuse, while deprivation was operationalized as emotional neglect (Hoffman et al., 2019). We measured household abuse using the adolescent report of the mean conflict subscale of the Family Environment Scale (FES), which consists of nine items targeting conflict in the family including family members arguing and fighting with each other. A higher score on the FES indicates more perceived physical abuse within the family (Moos & Moos, 1994). The FES has been found to have a Cronbach’s alpha of 0.68 (Zucker et al., 2018) and acceptable external validity (Moos, 1990). Parental acceptance was measured using the adolescent report of the mean acceptance subscale of the Children’s Report on Parent Behavior (CRPBI), which consists of five items measuring the care, support, and emotion regulation provided the primary caregiver. A higher score on the CRPBI indicates less perceived emotional neglect (Schaefer, 1965). The CRPBI has a Cronbach’s alpha of 0.71 (Zucker et al., 2018) and acceptable interrater reliability (Schwarz, Barton-Henry, & Pruzinsky, 1985). These measures were selected for use by the ABCD study due to their brevity and validity across different cultures represented in the US (Zucker et al., 2018). However, the internal consistency of the FES and CRPBI may suggest that they include items measuring heterogeneous constructs or with moderate inter-relatedness (Bland & Altman, 1997).

Previous studies have shown that both family and neighborhood SES indicators are associated with ELA exposure and brain development (Norbom et al., 2022; Ziegler et al., 2020), and we therefore included total family income (scored as 10 ordinal categories), education of the primary caregiver (scored as 21 ordinal categories), and the area deprivation index (Kind et al., 2014; ADI, scored in percentiles) as covariates in sensitivity analyses.

### MRI acquisition and processing

The ABCD study performs multimodal MRI (Casey et al., 2018), including T1w and T2w MRI. These sequences were acquired with harmonized parameters, and using real time motion correction where available, across Siemens, General Electric or Philips 3T scanner platforms (Casey et al., 2018). The full processing pipeline is described in detail elsewhere (Hagler et al., 2019). Briefly, the T1w and T2w images are corrected for gradient nonlinear distortion, coregistered, and processed using FreeSurfer v 7.1.1. We extracted cortical thickness, cortical surface area, and gray matter signal intensities from T1w and T2w images for 34 regions of the Desikan–Killiany atlas. Gray matter signal intensities were extracted 0.2 mm away from the gray–white matter border. We also extracted the volumes, T1w, and T2w signal intensities from seven subcortical gray matter regions (amygdala, hippocampus, putamen, pallidum, caudate nucleus, thalamus, nucleus accumbens) obtained from the FreeSurfer’s aseg atlas. The gray matter T1w/T2w ratio was calculated by dividing the T1w and T2w signal intensities per anatomical region (Glasser & Van Essen, 2011). We thereafter calculated the mean value for each measure across both hemispheres for each region to reduce the number of dependent variables. Only participants where both the T1w and T2w images passed the ABCD study’s standardized quality control were included in the analyses (Casey et al., 2018; Hagler et al., 2019).

### Statistical analysis

We used linear mixed-effects models (LMEs) with maximum likelihood estimation (lme4 version 1.1-29 and LmerTest version 3.1-3) in R (version 4.2.1) to test the association between brain structure and ELA while adjusting for sex, age at first MRI, and 29 MRI scanners. Longitudinal models additionally include a main effect of time (MRI-derived measure at ages 9–11 vs. 11-3) and interaction effect(s) between the ELA variables and time. Analyses of subcortical volume and cortical surface area were additionally adjusted for intracortical volume. Random intercepts were fitted for each family to adjust for the clustering of siblings, and random intercepts for each participant were fitted in longitudinal models. P-values were adjusted for multiple comparisons using the false discovery rate (FDR). Cohen’s *d* and Pearson’s *r* effect sizes were estimated by standardizing model coefficients (effect size version 0.8.5). Finally, we estimated the correlation between numerical variables at ages 9–11 using Kendall rank correlations (Supplementary Table S1).

We first investigated the association between the number of experienced traumatic events and regional gray matter structure at baseline (ages 9–11) followed by modeling the association between emotional neglect and physical abuse and regional gray matter structure. The LME models were specified as: dependent variable ~ trauma variable(s) + baseline age + sex + MRI scanner + (1 | family). We then tested longitudinal associations by including data from both the baseline (ages 9–11) and follow-up MRI (at ages 11–13), including the main effect of the number of experienced traumatic events, main effect of time point, and the interaction between time and the number of traumatic events. The LME models were specified as: dependent variable ~ trauma variable(s) * time point + baseline age + sex + MRI scanner + (1 | participant) + (1 | family). Similar models were performed for parental acceptance and household abuse. As SES may confound the association between ELA and gray matter structure (Tooley et al., 2021), we also performed sensitivity analyses in which we additionally included total family income, education of the primary caregiver, and ADI at ages 9–11 as covariates. Finally, we compared trauma resilient and trauma susceptible versus those with no trauma exposure or an internalizing disorder by including being resilient or susceptible as dummy variables, making having no trauma exposure/internalizing disorder the reference category for both.

We excluded participants who did not pass the quality control procedure of the T1w or T2w images or where information on either demographic, ELA, or SES variables were missing, resulting in 8059 remaining participants at ages 9–11 years. For the analyses examining differences related to being trauma resilient or susceptible, we also excluded participants with missing diagnostic information or who had not experienced trauma but did have an internalizing diagnosis, resulting in 6414 participants at ages 9–11 years. Longitudinal analyses used the same exclusion criteria while also requiring T1w and T2w data at both time points resulted in 1923 participants, and 1538 participants when examining differences related to being exposed to trauma and having an internalizing diagnosis.

### Ethics

A centralized institutional review board approval of procedures was obtained from the University of California, San Diego. Written informed consent was obtained by parent or guardian, and assent from the participants, before partaking in the ABCD study. The current study was approved by the Regional Committees for Medical and Health Research Ethics South-East Norway (REK 2019/943).

## Results

### Participant characteristics

As described in Table 1, the mean age at baseline was 9.93 years (SD = 0.63, range 8.91–11.10) and 11.83 after 2 years (SD = 0.66, range = 10.58–13.83). Then, 3897 (48%) patients were female at baseline, while 903 (47%) patients were female after 2 years. The sample included participants from 6957 families at baseline and 1769 families at the 2-year follow-up. We also compared the included participants and participants who were excluded due to missing data at baseline (Table 1). The analyses revealed that the included participants were slightly younger, had a lower ADI, had experienced more parental acceptance and fewer traumas, and were less likely to be diagnosed with an internalizing disorder. Moreover, there were a higher percentage of White participants in the included versus excluded participants, and they generally had higher parental education and household income.

**Table 1.** Sociodemographic, clinical, and trauma-related characteristics in included and excluded participants at ages 9–11

	Included (*n* = 8,059)	Excluded (*n* = 3,817)	*p*-value
	**M (SD)**		
Age at baseline (Years)	9.93 (0.63)	9.89 (0.62)	0.005
Area deprivation index (%)	38 (26)	45 (29)	<0.001
Unknown	0	879	
Number of traumatic events (count)	0.48 (0.93)	0.53 (0.91)	0.013
CRPBI (mean score)	2.79 (0.30)	2.77 (0.32)	0.005
Unknown	0	33	
FES (sum score)	−0.03 (1.27)	−0.01 (1.34)	0.3
Race/ethnicity			<0.001
White	4,570 (57%)	1,610 (42%)	
Hispanic	1,565 (19%)	846 (22%)	
Black	933 (12%)	851 (22%)	
Other	830 (10%)	417 (11%)	
Asian	161 (2.0%)	91 (2.4%)	
Unknown	2	0	
	**N (%)**		
Sex			0.10
Male	4,164 (52%)	2,032 (53%)	
Female	3,897 (48%)	1,783 (47%)	
Primary caregiver education			<0.001
Bachelor’s degree (ex. BA)	2,437 (30%)	752 (24%)	
Master’s degree (ex. MA)	1,671 (21%)	533 (17%)	
Some college	1,285 (16%)	544 (17%)	
Associate degree: Occupational	584 (7.2%)	280 (9.0%)	
High school graduate	489 (6.1%)	308 (9.9%)	
Associate degree: Academic program	477 (5.9%)	164 (5.2%)	
Doctoral degree (ex. PhD)	287 (3.6%)	95 (3.0%)	
Professional school degree (ex. MD)	251 (3.1%)	86 (2.8%)	
GED or equivalent	174 (2.2%)	93 (3.0%)	
12th grade	102 (1.3%)	80 (2.6%)	
11th grade	89 (1.1%)	65 (2.1%)	
9th grade	78 (1.0%)	42 (1.3%)	
10th grade	51 (0.6%)	30 (1.0%)	
6th grade	35 (0.4%)	20 (0.6%)	
8th grade	29 (0.4%)	17 (0.5%)	
7th grade	9 (0.1%)	6 (0.2%)	
5th grade	4 (<0.1%)	5 (0.2%)	
3rd grade	4 (<0.1%)	2 (<0.1%)	
4th grade	2 (<0.1%)	2 (<0.1%)	
1st grade	2 (<0.1%)	0 (0%)	
2nd grade	1 (<0.1%)	0 (0%)	
Unknown	0	691	
Household income			<0.001
$100,000–$199,999	2,683 (33%)	685 (30%)	
$75,000–$99,999	1,153 (14%)	310 (13%)	
$50,000–$74,999	1,046 (13%)	315 (14%)	
$200,000 and greater	1,065 (13%)	293 (13%)	
$35,000–$49,999	671 (8.3%)	168 (7.3%)	
$25,000–$34,999	450 (5.6%)	156 (6.7%)	
$16,000–$24,999	322 (4.0%)	128 (5.5%)	
Less than $5,000	247 (3.1%)	102 (4.4%)	
$5,000–$11,999	241 (3.0%)	98 (4.2%)	
$12,000–$15,999	183 (2.3%)	58 (2.5%)	
Unknown	0	1,502	
Trauma exposure			0.067
No traumatic experience	5,287 (66%)	2,436 (64%)	
One or more traumatic experiences	2,774 (34%)	1,379 (36%)	
Internalizing disorder			0.12
No internalizing disorder	5,258 (66%)	2,426 (65%)	
Internalizing disorder	2,695 (34%)	1,326 (35%)	
Unknown	108	63	
Trauma exposure and internalizing disorder			0.005
Neither trauma exposure nor internalizing disorder	3,651 (46%)	1,691 (45%)	
Trauma exposure and no internalizing disorder	1,607 (20%)	735 (20%)	
No trauma exposure and internalizing disorder	1,537 (19%)	684 (18%)	
Trauma exposure and internalizing disorder	1,158 (15%)	642 (17%)	
Unknown	108	63	

### Association between ELA and gray matter structure at ages 9–11

**Figure 1.**
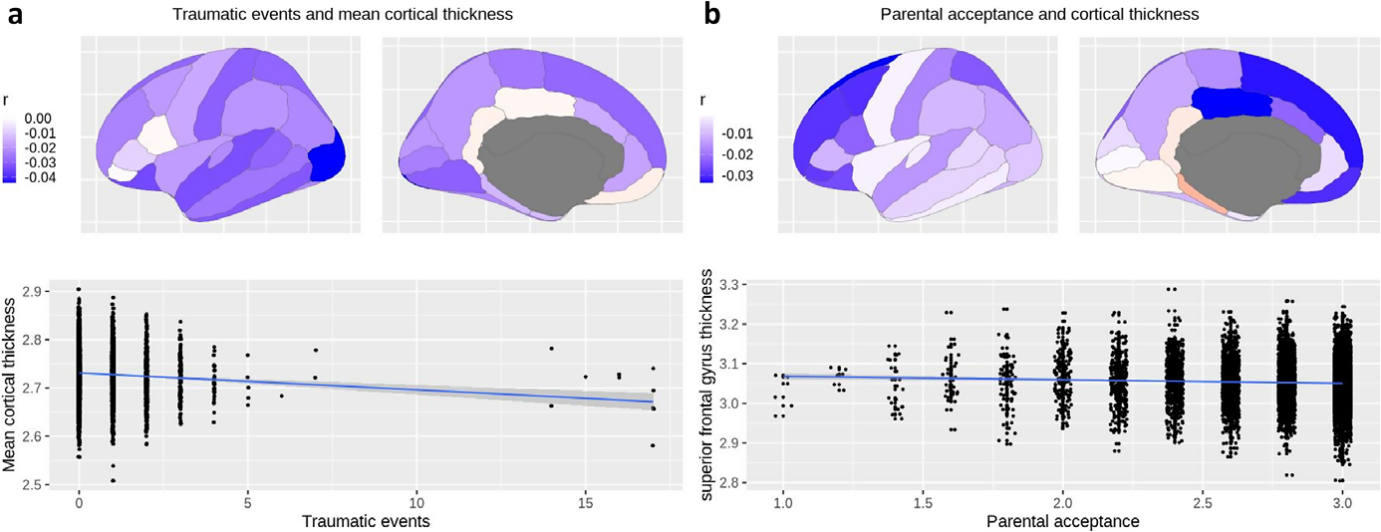
Associations between trauma exposure, parental acceptance, and cortical thickness at ages 9–11 (N = 8059). *Note:* (a) The number of experienced traumatic events is associated with thinner cortical thickness, particularly in the prefrontal cortex and posterior cingulate. (b) Greater parental acceptance is associated with thinner cortical thickness, particularly in the superior frontal, medial orbitofrontal, and posterior cingulate cortex.

LMEs revealed that more trauma exposure was significantly associated with lower levels of mean cortical thickness (*r* = −0.03, *t* = −3.20, FDRp = 0.01) (Table 2, Figure 1), while higher levels of parental acceptance were associated with thinner posterior cingulate, superior frontal, orbitofrontal, caudal, and rostral middle frontal cortices (Table 2, Figure 1). We found no significant associations between trauma exposure, parental acceptance or household abuse and baseline cortical surface area, T1w/T2w ratio, or subcortical volume.

**Table 2.** Main associations between and gray matter microstructure and macrostructure

ELA variable	Modality and brain region	Effect size			
Association between ELA and brain structure at ages 9–11		*r*	95% CI	*t*	FDRp
Number of traumatic events	Mean cortical thickness	–0.034	–0.055 – –0.013	–3.203	0.019
Parental acceptance	Thickness posterior cingulate cortex	–0.035	–0.026 – –0.056	–3.153	0.031
Parental acceptance	Thickness superior frontal gyrus	–0.033	–0.015 – –0.055	–3.124	0.031
Parental acceptance	Thickness medial orbitofrontal gyrus	–0.031	–0.015 – –0.052	–2.917	0.041
Parental acceptance	Thickness caudal middle frontal gyrus	–0.030	–0.011 – –0.051	–2.748	0.044
Parental acceptance	Thickness rostral middle frontal gyrus	–0.029	–0.015 – –0.050	–2.730	0.044

Associations between trauma exposure and internalizing diagnosis and brain structure at ages 9–11		*d*	95% CI	*t*	FDRp
Trauma but not internalizing diagnosis vs. neither trauma nor diagnosis	Mean cortical thickness	–0.091	–0.148 – –0.034	–3.132	0.015
Trauma and internalizing diagnosis vs. neither trauma nor diagnosis	Volume hippocampus	–0.089	–0.138 – –0.040	–1.849	0.003
Trauma and internalizing diagnosis vs. neither trauma nor diagnosis	Total cortical surface area	–0.046	–0.077 – –0.015	–2.865	0.035

Interaction between ELA at ages 9−11 and change in gray matter from ages 9–11 to 11−13		*d*	95% CI	*t*	FDRp
Parental acceptance	Thickness isthmus cingulate	–0.031	–0.049 – –0.013	–3.413	0.023
Household abuse	T1w/T2w nucleus accumbens	–0.057	–0.093 – –0.022	–3,189	0,019
Household abuse	T1w/T2w caudal anterior cingulate gyrus	–0.061	–0.098 – –0.025	–3.283	0.019
Household abuse	T1w/T2w rostral anterior cingulate gyrus	–0.064	–0.103 – –0.025	–3.248	0.019
Household abuse	T1w/T2w caudal anterior cingulate gyrus	–0.047	–0.077 – –0.017	–3.059	0.019
Household abuse	T1w/T2w caudal middle frontal gyrus	–0.047	–0.077 – –0.017	–3.078	0.019
Household abuse	T1w/T2w banks of the superior temporal gyrus	–0.047	–0.078 – –0.016	–2.976	0.021
Household abuse	T1w/T2w pars opercularis	–0.043	–0.072 – –0.014	–2.885	0.024
Household abuse	T1w/T2w transverse temporal gyrus	–0.044	–0.076 – –0.012	–2.733	0.030
Household abuse	T1w/T2w caudate nucleus	–0.039	–0.067 – –0.011	–2.729	0.030
Household abuse	T1w/T2w pars triangularis	–0.045	–0.078 – –0.011	–2.621	0.034
Household abuse	T1w/T2w superior temporal gyrus	–0.040	–0.069 – –0.010	–2.650	0.034
Household abuse	T1w/T2w supramarginal gyrus	–0.037	–0.066 – –0.009	–2.546	0.038

Abbreviations: CI = confidence interval, ELA = early life adversity, FDR = false discovery rate.

**Figure 2.**
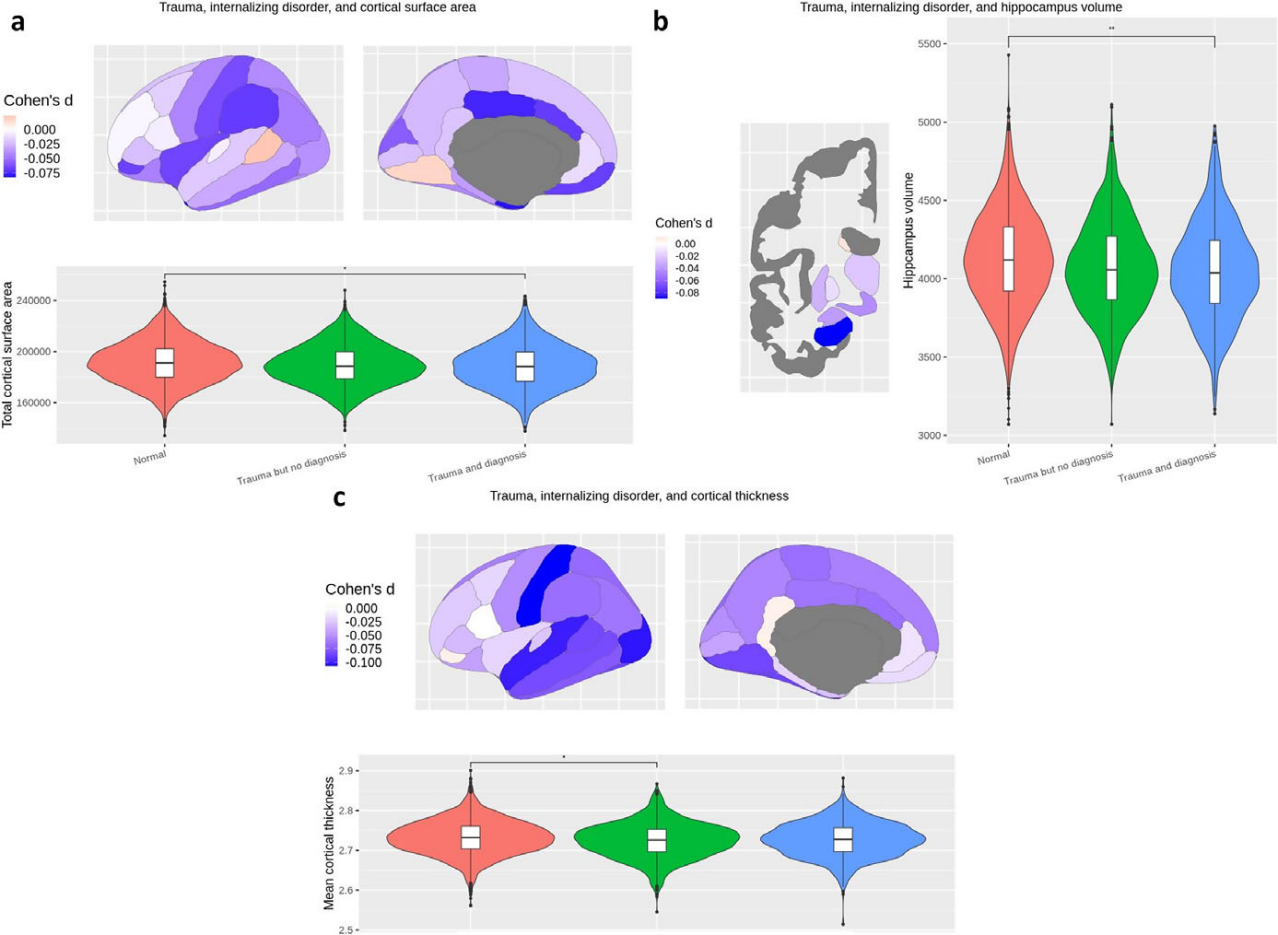
Association between trauma exposure, internalizing diagnosis, and gray matter macrostructure at ages 9–11 (N = 6414). *Note:* Participants with both trauma exposure and an internalizing diagnosis (*n* = 1158) showed less total cortical surface area (Panel a) and smaller hippocampus volume (Panel b) compared to participants with neither trauma exposure nor an internalizing diagnosis (*n* = 3650). Participants with trauma exposure but not an internalizing diagnosis (*n* = 1606) showed significantly thinner mean cortical thickness compared to compared to participants with neither trauma exposure nor an internalizing diagnosis (Panel c).

Trauma-resilient individuals showed lower mean cortical thickness (*d* = −0.09, *t* = −3.13, FDRp = 0.02), while trauma-susceptible individuals showed smaller hippocampus volume (*d* = −0.09, *t* = −1.9, FDRp < 0.01) and total cortical surface area (*d* = −0.05, *t* = −2.9, FDRp = 0.04), compared to those with neither trauma exposure nor an internalizing disorder (Table 2, Figure 2).

We performed sensitivity analyses that additionally adjusted for SES, including parental education, income, and ADI. The analyses revealed an attenuated association between trauma exposure and mean cortical thickness (*r* = −0.02, *t* = −1.87, FDRp = 0.38) after additionally adjusting for SES, while the associations between parental acceptance and cortical thickness in the posterior cingulate, superior frontal, orbitofrontal, caudal, and rostral middle frontal cortices remained significant. Interestingly, and an association between parental acceptance and mean cortical thickness became significant (*r* = −0.03, *t* = −2.50, FDRp = 0.04) after additionally adjusting for SES. Please see Supplemental Material for SES-adjusted analyses and uncorrected p-values. The association between trauma resiliency/susceptibility and mean cortical thickness, hippocampus volume, and total cortical surface area became insignificant after adjusting for SES.

### Changes in gray matter structure from ages 9–11 to 11–13

LMEs revealed that cortical thickness decreased over time for all brain regions, while cortical surface area showed both time-dependent increases and decreases. Similarly, there were significant changes in subcortical volume over time in all regions (indicating both increases and decreases). Finally, we found significant increases in T1w/T2w ratio over time in most regions, except for significant decreases in the temporal pole, nucleus accumbens, hippocampus, middle temporal gyrus, inferior temporal gyrus, and medial orbitofrontal gyrus (*d* ≥ −0.04, *t* ≥ −2.16, FDRp ≤ 0.04. These findings were unaffected by adjusting for SES in sensitivity analyses (see Supplemental Material for complete results and SES-adjusted analyses).

### Influence of ELA at ages 9–11 on subsequent changes in gray matter structure from 9–11 to 11–13

**Figure 3.**
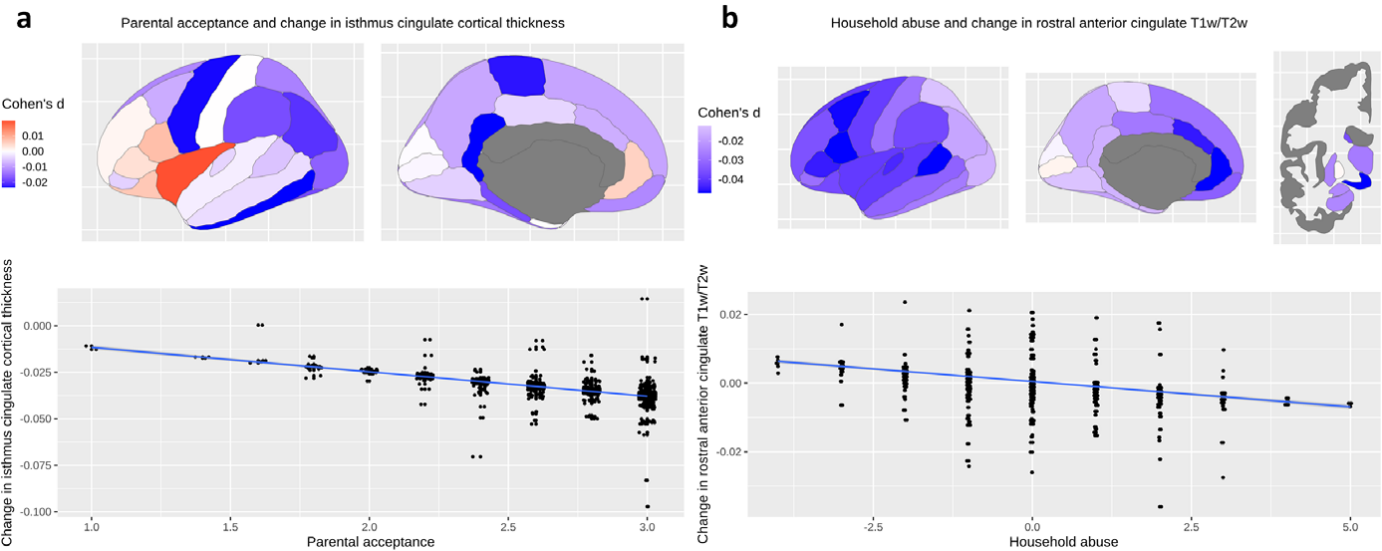
Association between parental acceptance or household abuse at ages 9–11 and change in cortical thickness and T1w/T2w from ages 9–11 to 11–13 (N = 1923). *Note:* (a) Parental acceptance at ages 9–11 was associated with decreased isthmus cingulate cortical thickness at ages 11–13. (b) Household abuse was associated with decreased rostral anterior cingulate T1w/T2w at ages 11–13.

LMEs revealed a significant interaction between parental acceptance at baseline and change over time in cortical thickness of isthmus cingulate (*d* = −0.03, *t* = −3.42, pFDR = 0.02), indicating that more parental acceptance at baseline was associated with more thinning of the isthmus cingulate cortex over time (Table 2, Figure 3a). We also found a significant interaction between household abuse at baseline and change over time in T1w/T2w ratio (*d* ≥ 0.04, *t* ≥ −2.55, pFDR = 0.04) in the nucleus accumbens, caudal anterior cingulate, rostral anterior cingulate, caudal middle frontal gyrus, banks of the superior temporal sulcus, pars opercularis, transverse temporal gyrus, caudate nucleus, precentral gyrus, pars triangularis, superior temporal gyrus, and supramarginal gyrus, indicating that more household abuse at baseline was associated with less change in T1w/T2w ratio over time (Table 2, Figure 3b).

We found no significant main effect or group by time interactions when comparing participants being resilient versus susceptible to trauma. All longitudinal results remained significant after additionally adjusting for SES.

## Discussion

ELA is associated with negative life outcomes, including increased risk for internalizing mental disorders (McLaughlin et al., 2019). Characterizing the effects of ELA on brain development in the transformative years of childhood and adolescence may help determine the biological mechanisms of stress susceptibility versus resilience. Using cross-sectional and longitudinal data from the largest brain imaging study in adolescence worldwide, we found that more trauma exposure, trauma resiliency, and more parental acceptance were associated with lower levels of mean cortical thickness at ages 9–11 years. Meanwhile, those who were trauma susceptible showed both smaller hippocampal volume and total cortical surface area. Longitudinal analyses revealed widespread decreased cortical thickness, increased cortical surface area, increased subcortical gray matter volume, and increased T1w/T2w ratio from ages 9–11 to 11–13 years, fitting with previously reported brain maturation patterns in youths (Grydeland et al., 2019; Herting et al., 2018; Norbom et al., 2021; Rutherford et al., 2022; Tamnes et al., 2017). Adolescents experiencing higher levels of baseline parental acceptance showed faster thinning of the cortex over time, while experiencing household abuse was associated with a slower increase in T1w/T2w ratio over time across several cortical and subcortical regions. Our findings support both the cumulative-risk and dimensional models, as both trauma exposure in general and more specific indices of threat and deprivation during childhood may influence the development of gray matter during adolescence (McLaughlin et al., 2019). Moreover, we are the first to show that ELA, and household abuse in particular, predicts a slower subsequent development of the T1w/T2s ratio, possibly reflecting slower cortical myelination.

Our cross-sectional findings of more trauma exposure being linked to widespread lower levels of cortical thickness fits well with previous meta-analyses of independent datasets (Pollok et al., 2022; Yang et al., 2023), as well as a previous report from the ABCD study (Jeong et al., 2021). Moreover, we found similar associations between a thinner cortex and greater caregiver emotional acceptance. The latter finding contradicts a recent study reporting an opposite pattern in a relatively small (*n* = 72) sample of younger participants (aged 5–10) (Machlin et al., 2023), while another recent study of 149 youths aged 8–17 reported no significant association between deprivation and cortical thickness (Peverill et al., 2023). The discrepant or nonsignificant findings in some previous studies (Machlin et al., 2023; Peverill et al., 2023) may reflect differences in how deprivation and neglect were measured, the age of the participants, and the severity of these experiences. Notably, most reported associations between different forms of ELA and brain structure are subtle and likely influenced by multiple interrelated variables (Beck et al., 2024; Jeong et al., 2021; Machlin et al., 2023; McLaughlin et al., 2019; Peverill et al., 2023). For example, both brain development and ELA may both be linked to factors such as SES (Rakesh, Whittle, Sheridan, & McLaughlin, 2023) or puberty (Beck et al., 2023; Colich et al., 2020), which can confound the direct association between ELA and brain development.

Uncovering if ELA is associated with accelerated or delayed brain maturation is an area of current scientific focus (Colich et al., 2020; McLaughlin et al., 2019). Indeed, cross-species models have suggested that earlier brain maturation may be an advantage in harsh environments where the child may have limited parental support (Luby, Baram, Rogers, & Barch, 2020). However, most previous studies have relied on cross-sectional data, which limits linking ELA and individual rates of brain maturation. This is particularly important when investigating gray matter microstructure and macrostructure, as developmental studies have reported that T1w/T2w ratio increases in the prefrontal, limbic and insular cortices accelerate around age 13 (Grydeland et al., 2019), while cortical thickness and surface area may start declining even earlier (Tamnes et al., 2017). The significant interaction between household abuse at ages 9–11 and time on the T1w/T2w ratio suggest that experiencing more household abuse is linked to slower maturation of T1w/T2w ratio over time (Grydeland et al., 2019; Norbom et al., 2021). Experiencing more parental acceptance and support were linked to a larger decrease in cortical thickness, in line with normative development (Tamnes et al., 2017). This is further supported through a recent report from the ABCD study measuring brain age using both structural, diffusion-weighted and resting-state functional MRI (Beck et al., 2024), where emotional neglect and family conflict were associated with a younger brain age, whereas SES, neighborhood safety, and caregiver psychopathology were related to an older brain age (Beck et al., 2024).

Motivated by recent studies reporting delayed hippocampal volumetric increase and prefrontal myelination in trauma-susceptible individuals (Hettwer et al., 2024; Keding et al., 2021); we also explored this interaction for gray matter macrostructure and microstructure. We found that trauma-susceptible individuals had smaller hippocampal volume and smaller cortical surface area at ages 9–11, but trauma susceptibility did not influence change in gray matter structure over time. The hippocampus is a complex and heterogeneous structure supporting many cognitive and emotional functions (Smolker et al., 2022). It also has an especially high density of cortisol receptors and is a key regulator of the hypothalamic–pituitary–adrenal axis (McLaughlin et al., 2019). As a result, the hippocampus may be particularly vulnerable to the neuroendocrine disturbances associated with ELA and experiencing internalizing disorder symptoms (McLaughlin et al., 2019). Moreover, due to its key role in several cognitive and emotional functions, developmental aberrations in hippocampal structure are likely to increase vulnerability to future mental health problems (Smolker et al., 2022). In addition to lower hippocampal volume, trauma-susceptible adolescents had smaller cortical surface area compared to normal developing youths. Cortical surface area has a different developmental trajectory and possibly different genetic underpinning compared to cortical thickness (Tamnes et al., 2017; van der Meer et al., 2020). While the development of cortical surface area has traditionally been linked to the organization of cortical columns, it may also be associated with the age-related increase in white matter fibers terminating in the cortex (Cafiero, Brauer, Anwander, & Friederici, 2019). While speculative, the findings of delayed T1w/T2w ratio development and lower surface area in trauma exposed and trauma-susceptible youths could suggest an effect of ELA on the trajectory of cortical myeloarchitectural development (Warhaftig et al., 2023). In contrast, being resilient to trauma was linked to a thinner cortex. This suggests that being resilient to trauma is associated with age-appropriate thinning of the cortex, possibly reflecting accelerated maturation, including faster synaptic pruning and dendritic arborization (Norbom et al., 2021; Tamnes et al., 2017).

Our study has several limitations. First, the measures of ELA in the ABCD study are limited in scope and how well they measure when ELA occurred. We defined trauma exposure using the 17-item post-traumatic stress module of the Kiddie-SADS-PL, which is filled out by the primary caregiver and does not specify when the trauma occurred (Kaufman et al., 1997). Further, it was difficult to disentangle if youths and parents agreed that a traumatic event had occurred. We used the youth’s own reports of household abuse and parental acceptance as these may more closely reflect the youth’s experience of ELA. However, these measures ask how the youth experience their environment at the time of inclusion, which could mask previous experiences where the environment was better or worse. The divergence between null findings for parent reported trauma exposure but positive results for youth reported household abuse and parental acceptance may be explained by the subjective nature of the youth reports. Indeed, previous studies suggest that adverse effects of ELA are primarily driven by the subjective experience of the ELA, rather than external or objective trauma exposure (Francis et al., 2023). Notably, the ABCD study has a limited number of variables assessing threat and deprivation, making us unable to investigate if similar associations with brain development emerge with alternative measures. This also applies to other possible confounding variables such as nutrition, physical activity, prenatal exposures to alcohol, drugs, or toxins, and more (Agarwal et al., 2025). However, we believe some of these confounding variables are adjusted for in the present manuscript through the ADI that includes multiple measures related to socioeconomic adversity (Kind et al., 2014). We also found that adjusting for SES attenuated our cross-sectional findings, in particular the associations between trauma susceptibility/resiliency and brain morphometry at ages 9–11. Notably, the longitudinal, findings remained significant after adjusting for SES. This suggests that the extent to which trauma susceptibility/resiliency influence brain morphometry in late childhood could depend on the family’s SES, which is an area of ongoing research (Rakesh et al., 2023). We also showed that the excluded participants (based on incomplete data) had slightly higher SES and less exposure to ELA, which should be considered when generalizing the findings of the study. Similarly, it is unclear how generalizable findings from a US study are to other national and socioeconomic contexts (Xu et al., 2024). Our longitudinal sample size was also limited due to using the Annual Release 4.0 of the ABCD study and requiring complete data for covariates of interest. Although our study is one of the largest on ELA and adolescent brain development to date, future studies should make use of more participants and time points as they become available in new data releases. Indeed, new data releases in the ABCD study would allow for investigating how ELA experienced after ages 9–11 and the impact of cumulate adverse experiences, including of how ELA in combination with later traumatic experience may lead to the development of PTSD (Gould et al., 2021). Since some participants had not yet reached the second MRI at the time of the current data release, we could not reliably determine if the lack of longitudinal MRI data was related to attrition or not. However, there is evidence that having a stay-at-home parent is associated with missing the 2-year follow-up in the ABCD study, while greater traveling distance, having married parents, or Asian ethnicity is associated with late visits at the 2-year follow-up (Feldstein Ewing et al., 2022). Given current and previous findings (Beck et al., 2024) relating ELA to longitudinal brain development, future studies should consider using normative models to better understand how ELA may drive deviations from expected developmental trajectories (Wong et al., 2023). Future studies may also investigate possible sex differences in the association between ELA and brain development, as previous findings suggest that sex may moderate the association between ELA and gray matter volume (Kelly et al., 2015) and may interact with age in the development of T1w/T2w ratio in adolescence (Norbom et al., 2020).

Finally, the underlying biology of T1w/T2w ratio is debated. Early studies have referred to T1w/T2w ratio as ‘myelin mapping’ (Glasser & Van Essen, 2011), whereas more recent studies have also reported associations between T1w/T2w ratio and neuronal density, axonal diameter as well as other tissue properties (Boroshok et al., 2023; Norbom et al., 2021). Thus, future studies should also investigate associations between ELA and other myelin-sensitive MRI sequences. Our study is strengthened by its large sample size and longitudinal MRI, making it possible to disentangle normal and abnormal patterns of gray matter maturation.

In conclusion, we found that gray matter macrostructure, including cortical thickness, is negatively associated with trauma exposure and parental acceptance at ages 9–11. The impact of trauma exposure on hippocampal volume and cortical surface area was further aggravated by having an internalizing disorder, while being resilient to trauma was linked to an age-appropriate thinning of the cortex. Greater parental acceptance was associated with faster thinning of cortical thickness from ages 11 to 13. In addition, being exposed to household abuse was associated with widespread slowing of the T1w/T2w ratio development, possibly reflecting delayed myelination. Our findings suggest that different ELA indices are distinctly associated with the development of gray matter macrostructure and microstructure, and that longitudinal analyses are crucial for disentangling normal and abnormal brain development during adolescence.

## Supporting information

Thorsen et al. supplementary material Thorsen et al. supplementary material

## Supplementary material

The supplementary material for this article can be found at http://doi.org/10.1017/S0033291725102651.
